# Subsequent pregnancy: healing to attach after perinatal loss

**DOI:** 10.1186/1471-2393-15-S1-A15

**Published:** 2015-04-15

**Authors:** Joann O’Leary

**Affiliations:** 1 Center for Early Education and Development, University of Minnesota, MN, USA

## 

The paralysing feelings of grief after perinatal loss can cause an overwhelming feeling of being abandoned, creating confusion, disorientation and hopelessness, altering future pregnancies for parents, children alive at the time and those that follow. Research with parents’ pregnant following loss, elderly bereaved parents with no intervention, and children born after loss, some now adults, show the importance of guided intervention in the pregnancy that follows.

Parents who suffered losses fifty or more years ago were offered little guidance or rituals in healing [1], followed doctors’ advice and, even those that requested to see their baby were told it was for the best if they didn’t. The message was to “buck up,” get pregnant again, frequently living a life time in the shadow of the experience. Parents’ unresolved grief and tendency to not share what happened often became an emotional burden carried by siblings into adulthood [2-4].

The professional’s goal is not to sever parents bond with the deceased baby but to help them create memories that recognize the psychological/spiritual dimensions of the relationship that does not end [5] to integrate the deceased baby into their lives [5,7] as they embrace a new unborn baby; important in lieu of the research that fear of loss can hinder attachment to the child born after [6,8].

A framework for understanding pregnancy loss and the pregnancy that follows is integration of the models of attachment and loss that honours their parenting relationship with both the deceased baby and new unborn baby [7]. Research in maternal fetal medicine and prenatal psychology suggests there is a deep connection developing during pregnancy, maternal/fetal programming occur in parallel [9-11] and are bidirectional [11,12]. Prenatal diagnostics, genetic screening, and fetal surgery have changed the medical and cultural status of the maternal-fetal relationship, suggesting attachment begins at an earlier stage. Investment is a more active process of involvement in the pregnancy [13] whereas attachment is concerned with the development of feelings for the baby as the parent seeks: to know, to be with and interact with, to protect, to avoid separation or loss and to gratify needs of the unborn child [14]. It is not just prenatal caregiving [15] but developing a relationship as unresolved histories of early relational trauma or loss often remain actively dysregulated in the intra-psychic mind of a parent, becoming a powerful source for some prenate’s stress [10]. A prenatal attachment framework alters representations of the unborn child in parental behaviors using the message that “the baby is already here” while sustaining a continued bond to the deceased baby [7] as a family member in order to attach to the child that follows.

Parents’ need information on how to tell surviving children about their deceased sibling. Children need to know it’s okay to cry, be given appropriate information at their developmental age, reminded it’s not their job to take care of the parent, involved in family rituals, find a meaningful symbol to connect, and someone who will listen to their feelings [16]. Adults who were a subsequent child and research with parents raising children after a loss all shared surprisingly common themes; sensitivity/nurturing to others, curious and sadness of not knowing sibling; a deep understanding of death and were not afraid to be present to grieving people [2,3]. The theme that was different reflected children whose parents had supportive intervention at the time of loss or in the pregnancy that followed who described feeling loved and overprotective verses adults whose parents lacked support; half felt loved and cherished while others felt invisible in their families [17]. Parents who have support and guidance are very intentional in raising their children [17,18]. Within the context of loss common patterns and reactions of grief emerge throughout the continuum of life as we all rework pieces of our grief. Reconciling and healing is a process, not an event.
